# Neurological Outcomes Following Surgical Resection of Intradural Extramedullary Spinal Tumors: A Retrospective Analysis Using the American Spinal Injury Association (ASIA) Impairment Scale

**DOI:** 10.7759/cureus.99857

**Published:** 2025-12-22

**Authors:** Reem Samir Abboud, Gill E Sviri

**Affiliations:** 1 Neurosurgery, The Ruth and Bruce Rappaport Faculty of Medicine, Technion - Israel Institute of Technology, Haifa, ISR; 2 Neurosurgery, Rambam Medical Center, Haifa, ISR

**Keywords:** asia impairment scale, clinical neurosurgery, intradural extramedullary spinal, neurological outcomes, neurosurgery oncology, oncology, spinal tumors, spine surgery, surgery, neurology

## Abstract

Objective

Intradural extramedullary (IDEM) tumors, predominantly meningiomas and schwannomas, frequently require surgical resection. However, reliable predictors of postoperative neurological outcomes remain poorly defined. This study aimed to identify risk factors for neurological deterioration following IDEM tumor resection and to evaluate outcomes using the American Spinal Injury Association (ASIA) Impairment Scale.

Methods

A retrospective chart review was conducted of 93 consecutive patients who underwent surgical resection of IDEM tumors (52 meningiomas, 41 schwannomas) at a single tertiary neurosurgical center between 2008 and 2019. Patient demographics, tumor characteristics, perioperative variables, and medical comorbidities were systematically analyzed. The primary outcome was change in ASIA score from admission to last follow-up. Statistical analyses included paired t-tests, chi-square tests, Spearman correlations, and multivariable comparisons to identify potential risk factors.

Results

The cohort comprised 38 males and 55 females with a mean age of 59.8 ± 13.2 years. Mean operative duration was 142.2 ± 50.1 minutes. Median follow-up was 5 months. Mean ASIA score improved significantly from 4.14 ± 0.84 at admission to 4.74 ± 0.66 at follow-up (p < 0.001). Individual outcomes showed improvement in 49 patients (52.7%), stability in 43 (46.2%), and deterioration in 1 (1.1%). Notably, patients with obesity (p = 0.005) and a smoking history (p = 0.043) demonstrated significantly lower baseline ASIA scores but achieved comparable postoperative outcomes. No significant associations were identified between neurological outcomes and age, sex, tumor pathology, tumor location, symptom duration, operative duration, tumor size, or the presence of medical comorbidities.

Conclusions

Surgical resection of IDEM tumors demonstrates excellent neurological safety, with significant functional improvement in the majority of patients. The ASIA scale effectively captures neurological outcomes in this population. No modifiable perioperative risk factors for neurological deterioration were identified, supporting the safety of surgery across diverse patient populations.

## Introduction

Intradural extramedullary (IDEM) spinal tumors, comprising primarily meningiomas and nerve sheath tumors, represent a significant subset of adult spinal neoplasms and account for approximately one-third of all spinal cord tumors [1,2]. These lesions typically present with progressive pain, neurological deficits, and, in some cases, sphincter dysfunction, reflecting their mass effect on neural structures [3,4]. Advances in neuroimaging and intraoperative neurophysiological monitoring (IONM) have facilitated earlier diagnosis and safer surgical intervention, with gross total resection being the standard of care for most benign IDEM tumors [1,2,5].

Surgical excision of IDEM tumors is associated with high rates of neurological improvement and pain reduction, as well as durable gains in patient-reported quality of life [3,4]. However, the risk of postoperative neurological complications remains a concern, particularly for tumors located in the thoracic spine or those with anterior/ventral positioning, which are associated with higher rates of surgical morbidity [5,6]. Intraoperative neuromonitoring, while widely adopted, demonstrates high sensitivity and specificity for predicting new deficits, but its impact on long-term outcomes and extent of resection is variable and does not supplant clinical judgment [1,5,7].

Despite the overall favorable prognosis, the identification of reliable preoperative and perioperative risk factors for neurological deterioration remains an area of ongoing investigation. Current evidence suggests that preoperative neurological status and tumor location are the most consistent predictors of outcome, while other factors such as age, comorbidities, and operative duration have not shown significant associations [1,5,6]. This study aims to further characterize postoperative neurological outcomes and elucidate risk factors for deterioration following IDEM tumor resection, thereby informing surgical decision-making and perioperative management.

Previous studies on neurological outcomes and risk factors in patients with intradural spinal tumors have several key limitations regarding the use of neurological assessment scales such as the McCormick, Karnofsky, and American Spinal Injury Association (ASIA) scales. Most notably, the McCormick and Karnofsky scales are broad functional measures that lack sensitivity to subtle changes in neurological status and do not comprehensively capture specific motor, sensory, and autonomic deficits. This can lead to underestimation of postoperative neurological complications and may obscure the identification of granular risk factors for deterioration or recovery [8-11].

The ASIA scale, while more detailed and validated for spinal cord injury, has been underutilized in prior studies of intradural tumor surgery. As a result, previous investigations often relied on less standardized or less sensitive outcome measures, limiting comparability and the ability to construct robust predictive models for neurological complications [9,12,13]. Furthermore, many studies have used physician-rated scales without incorporating patient-reported outcomes, which can miss important aspects of functional recovery and quality of life [11].

These limitations affect interpretation by potentially overestimating the safety of surgery and underestimating the true incidence and spectrum of neurological complications. They also hinder the identification of specific risk factors, as broad scales may not detect associations with subtle preoperative deficits, tumor location, or perioperative events [9,10,14]. In contrast, the current investigation’s use of the ASIA scale allows for more precise quantification of neurological outcomes and risk factor analysis, improving the validity and clinical applicability of its predictive model [12,13].

Study rationale and objectives

IDEM spinal tumors, including meningiomas and schwannomas, are frequently managed with surgical resection, which generally yields favorable neurological and functional outcomes. However, a subset of patients experience postoperative neurological complications, and current literature demonstrates that reliable preoperative risk stratification remains limited [8-12]. Existing studies have identified factors such as preoperative neurological status, tumor location, size, and extent of resection as potential predictors, but consensus on their relative importance and integration into predictive models is lacking [9-14].

The rationale for this retrospective study is to address the clinical need for an evidence-based, quantitative approach to predicting neurological complications following IDEM tumor surgery. By systematically analyzing patient demographics, tumor characteristics, operative variables, and perioperative factors, this study aims to develop a predictive model that can be used to estimate individual risk profiles. The ASIA Impairment Scale will be employed as a standardized, validated measure of neurological function, enabling objective assessment of outcomes and facilitating comparison across studies [13,15].

The primary objectives are: (1) to identify preoperative and perioperative risk factors associated with postoperative neurological deterioration, (2) to construct and internally validate a predictive model for neurological complications using multivariate analysis, and (3) to assess the utility of the ASIA scale in quantifying neurological outcomes in this patient population. Secondary objectives include evaluating the impact of IONM and surgical approach on complication rates [8,12,14].

The significance of this study lies in its potential to improve clinical practice by providing a robust, data-driven tool for risk stratification and patient counseling. Predictive modeling has been shown to enhance surgical decision-making, optimize perioperative management, and inform shared decision-making in neuro-oncology [16-19]. By integrating the ASIA scale and leveraging retrospective cohort data, this study will contribute to the development of standardized outcome reporting and facilitate future prospective validation. Ultimately, the findings may support individualized treatment planning and improve neurological outcomes for patients undergoing IDEM tumor resection [8-17].

## Materials and methods

Study design and patient selection

**Figure 1:**
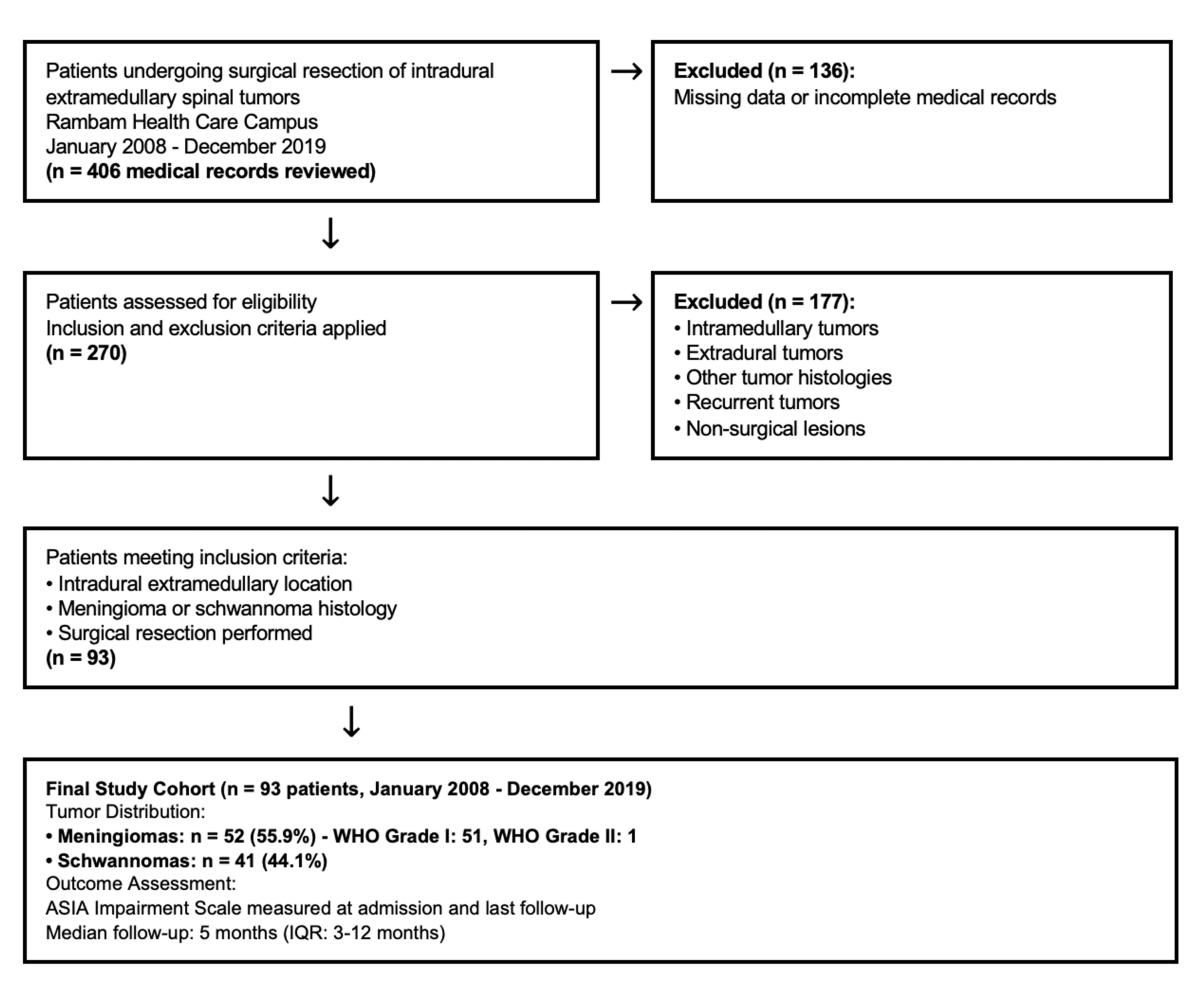
Patient selection flow chart. Patient selection flow chart illustrating the inclusion and exclusion criteria for the study cohort. A total of 93 patients with intradural extramedullary spinal tumors (52 meningiomas, 41 schwannomas) underwent surgical resection at Rambam Health Care Campus between January 2008 and December 2019. ASIA: American Spinal Injury Association.

We retrospectively reviewed the records of patients who underwent IDEM tumor resection at the Department of Neurosurgery, Rambam Health Care Campus, Haifa, Israel, between January 2008 and December 2019. Patients were identified using ICD-9 codes 2253 (benign neoplasm of spinal cord), 2254 (benign neoplasm of spinal meninges), and 2375 (neoplasm of uncertain behavior of spinal cord and brain). The initial database query yielded 406 records. After excluding 136 cases with insufficient documentation and 177 cases that did not meet inclusion criteria (non-IDEM tumors, brain tumors miscoded as spinal, no surgical intervention), 93 patients remained for analysis. The patient selection process and final cohort composition are illustrated in Figure 1.

Data collection

Clinical data were extracted from electronic medical records, including hospital admission and discharge summaries, operative reports, pathology reports, and radiological imaging studies. Collected variables included patient demographics (age, sex), medical comorbidities (cardiac disease, anticoagulation use, diabetes mellitus, hypertension, neurological conditions, pulmonary disease, hypothyroidism, obesity, smoking history, hyperlipidemia), tumor characteristics (location, size by pathological measurement, pathology), operative details (duration, intraoperative monitoring results, complications), and hospitalization data (length of stay, discharge destination).

Outcome measures

The primary outcome was change in neurological status assessed using the ASIA Impairment Scale at admission and last follow-up [20]. This scale was selected for the current study due to (1) ease of application for both research purposes and clinical patient assessment, and (2) utility in prognostic interpretation based on study results. The ASIA scale grades neurological status from A to E, as follows (Table 1). Follow-up duration ranged from 1 to 36 months (median 5 months, IQR 3-12 months).

**Table 1:** ASIA Impairment Scale Classification. Classification system based on the International Standards for Neurological Classification of Spinal Cord Injury (ISNCSCI) [20]. The ASIA scale grades neurological function from A (complete injury; no motor or sensory function preserved in the sacral segments S4-S5) to E (normal motor and sensory function). This scale has been validated for neurological assessment in spinal tumor patients. However, this classification system has inherent limitations: it does not recognize subtle motor changes (for example, the difference between muscle grade 3 and 4), nor does it identify isolated sensory impairment without accompanying motor dysfunction. ASIA: American Spinal Injury Association.

Grade	Classification	Definition
A	Complete	No motor or sensory function is preserved in the sacral segments S4-S5.
B	Incomplete	Sensory function is preserved below the neurological level and includes the sacral segments S4-S5, but no motor function is preserved more than three levels below the motor level.
C	Incomplete	Motor function is preserved below the neurological level, and more than half of key muscles below the neurological level have a muscle grade less than 3 (grades 0-2).
D	Incomplete	Motor function is preserved below the neurological level, and at least half of key muscles below the neurological level have a muscle grade ≥3.
E	Normal	Motor and sensory function are normal in all segments.

Statistical analysis

Descriptive statistics included means, standard deviations, medians, and interquartile ranges. Normal distribution was assessed using the Kolmogorov-Smirnov test. Continuous variables were compared using independent samples t-test or Mann-Whitney U test, as appropriate. Categorical variables were analyzed using chi-square test or Fisher’s exact test. Changes in ASIA scores from admission to follow-up were assessed using paired t-test. Correlations between ASIA score change and continuous variables (age, tumor size, operative duration, hospitalization length) were examined using Spearman correlation coefficient. Statistical significance was set at p < 0.05. All analyses were performed using SPSS version 27.

Ethical approval

This study was approved by the Rambam Health Care Campus Institutional Review Board (approval number: RMB-0029-21) in accordance with the Declaration of Helsinki. Given the retrospective nature and use of de-identified data, the requirement for informed consent was waived.

## Results

Patient demographics and baseline characteristics

A total of 93 patients underwent surgical resection of intradural extramedullary spinal tumors during the study period. The mean age at surgery was 59.8 ± 13.2 years (range: 18-84 years), with 38 males (40.9%) and 55 females (59.1%). Preoperative neurological status assessed by ASIA Impairment Scale classification revealed: grade A in 2 patients (2.2%), grade B in 1 patient (1.1%), grade C in 12 patients (12.9%), grade D in 45 patients (48.4%), and grade E in 33 patients (35.5%). The median tumor volume, based on pathological measurements, was 2.62 cm³ (range: 1.5-6.33 cm³).

Symptom duration was documented for 89 of 93 patients (95.7%): 1 patient (1.1%) presented with symptoms lasting ≤1 month, 38 patients (42.7%) had symptoms lasting 1 month to 1 year, and 50 patients (56.2%) had symptoms lasting >1 year (Table 2).

**Table 2:** Patient demographics, baseline clinical status, and preoperative characteristics (N = 93). Symptom-duration data were available for 89 of 93 patients (95.7%). All other baseline characteristics represent the complete cohort of 93 patients. ASIA Impairment Scale grades are presented as documented at hospital admission. ASIA: American Spinal Injury Association.

Characteristic	Value
Age at surgery, years	
Mean ± SD	59.8 ± 13.2
Range	18-84
Sex, n (%)	
Male	38 (40.9)
Female	55 (59.1)
Preoperative ASIA Impairment Scale score, n (%)	
A (Complete)	2 (2.2)
B (Incomplete - sensory only)	1 (1.1)
C (Incomplete - motor <3)	12 (12.9)
D (Incomplete - motor ≥3)	45 (48.4)
E (Normal)	33 (35.5)
Tumor size by pathological assessment, cm³	
Median (range)	2.62 (1.5-6.33)
Duration of symptoms (n = 89), n (%)	
Up to 1 month	1 (1.1)
1 month to 1 year	38 (42.7)
>1 year	50 (56.2)
Clinical presentation at admission	
Motor weakness, n (%)	59 (63.4)
Mean strength grade below injury level (1-5)	3.84 ± 1.13
Negative sensory symptoms, n (%)	32 (34.4)
Positive sensory symptoms, n (%)	56 (60.2)
Local pain, n (%)	60 (64.5)
Sphincter dysfunction, n (%)	12 (12.9)
Admission type, n (%)	
Elective	78 (83.9)
Transfer from another facility	3 (3.2)
Emergency	12 (12.9)

Clinical presentation at admission included motor weakness in 59 patients (63.4%), with mean muscle strength of 3.84 ± 1.13 (on a 1-5 scale) below the level of the lesion, negative sensory symptoms in 32 patients (34.4%), positive sensory symptoms in 56 patients (60.2%), local pain in 60 patients (64.5%), and sphincter dysfunction in 12 patients (12.9%). Admission type was elective in 78 patients (83.9%), hospital transfer in 3 patients (3.2%), and emergency admission in 12 patients (12.9%).

Perioperative data

Mean operative duration was 142.2 ± 50.1 minutes (range: 53-344 minutes). Among the 60 patients with intraoperative neuromonitoring, decreased electrophysiological signals were observed before surgery in 16 patients (26.7%), during surgery in 14 patients (23.3%), at the conclusion of surgery in 10 patients (16.7%), while 20 patients (33.3%) showed no signal changes at any time point throughout the procedure (Table 3).

**Table 3:** Perioperative and hospitalization data (N = 93). Signal changes were categorized by timing: before surgery (n = 16, 26.7%), during surgery (n = 14, 23.3%), at conclusion (n = 10, 16.7%), or no change throughout the procedure (n = 20, 33.3%). Categories are mutually exclusive.

Variable	Value
Operative duration, minutes	
Mean ± SD	142.2 ± 50.1
Range	53-344
Intraoperative complications, n (%)	1 (1.1)
Intraoperative electrophysiological monitoring results (n = 60), n (%)	
Decreased signals before surgery	16 (26.7)
Decreased signals during surgery	14 (23.3)
Decreased signals at end of surgery	10 (16.7)
No signal changes	20 (33.3)
Discharge destination, n (%)	
Home	67 (72.0)
Acute rehabilitation facility	25 (26.9)
Continued acute hospitalization	1 (1.1)
Hospital length of stay, days	
Preoperative, median (IQR; range)	1 (1-1; 0-20)
Postoperative, median (IQR; range)	4 (3-6; 1-17)
Total, median (IQR; range)	5 (4-7; 2-23)
Non-surgical medical complications, n (%)	
Wound infection	0 (0.0)
Cerebrospinal fluid leak	1 (1.1)
Deep vein thrombosis	2 (2.1)
Pulmonary embolism	2 (2.1)
Pleural effusion	0 (0.0)
Fever	1 (1.1)
No complications	87 (93.5)
Pathological diagnosis, n (%)	
Meningioma, WHO grade 1	51 (54.8)
Meningioma, WHO grade 2	1 (1.1)
Schwannoma (neurinoma)	41 (44.1)

Discharge disposition was home in 67 patients (72.0%), acute rehabilitation in 25 patients (26.9%), and continued hospitalization in 1 patient (1.1%). Median preoperative hospitalization was 1 day (IQR 1-1; range: 0-20 days), median postoperative hospitalization was 4 days (IQR 3-6; range: 1-17 days), and median total hospitalization was 5 days (IQR 4-7; range: 2-23 days).

Non-surgical medical complications included cerebrospinal fluid leak in 1 patient (1.1%), deep vein thrombosis in 2 patients (2.1%), pulmonary embolism in 2 patients (2.1%), and fever in 1 patient (1.1%). No wound infections or pleural effusions were recorded.

Pathological diagnosis revealed 51 WHO grade 1 meningiomas (54.8%), 1 WHO grade 2 meningioma (1.1%), and 41 schwannomas (44.1%).

Medical comorbidities

The most common comorbidities were hypertension in 52 patients (55.9%) and hyperlipidemia in 39 patients (41.9%). Other comorbidities included diabetes mellitus in 23 patients (24.7%), neurological disease in 22 patients (23.7%), anticoagulation use in 16 patients (17.2%), obesity in 14 patients (15.1%), smoking in 15 patients (16.1%), oncological history in 12 patients (12.9%), cardiac disease in 9 patients (9.7%), pulmonary disease in 8 patients (8.6%), hypothyroidism in 5 patients (5.4%), kidney disease in 3 patients (3.2%), rheumatic disease in 3 patients (3.2%), cerebrovascular disease in 1 patient (1.1%), and hyperthyroidism in 1 patient (1.1%). No patients had liver disease (Table 4).

**Table 4:** Medical comorbidities of study population (N = 93). Comorbidities are presented as number and percentage of the complete cohort (N = 93). Patients could have multiple comorbidities; therefore, categories are not mutually exclusive, and percentages sum to more than 100%.

Medical comorbidity	n (%)
Hypertension	52 (55.9)
Hyperlipidemia	39 (41.9)
Diabetes mellitus	23 (24.7)
Neurological comorbidities	22 (23.7)
Anticoagulant medication use	16 (17.2)
Smoking history (active or past)	15 (16.1)
Obesity	14 (15.1)
Oncological history	12 (12.9)
Cardiac disease	9 (9.7)
Pulmonary disease	8 (8.6)
Hypothyroidism	5 (5.4)
Kidney disease	3 (3.2)
Rheumatic disease	3 (3.2)
Cerebrovascular disease	1 (1.1)
Hyperthyroidism	1 (1.1)
Liver disease	0 (0.0)

Pathology by demographics

Meningiomas occurred predominantly in females (43/51, 84.3%), while schwannomas occurred predominantly in males (30/41, 73.2%) (p < 0.001). Mean age for meningiomas was 64.8 ± 9.8 years, significantly older than for schwannomas at 53.4 ± 14.2 years (p = 0.001) (Table 5).

**Table 5:** Tumor pathology stratified by patient demographics. Note: One patient had WHO grade 2 meningioma (excluded from this table).

	Meningioma grade 1 (n = 51)	Neurinoma (n = 41)	p-value
Age at surgery, years			
Mean ± SD	64.8 ± 9.8	53.4 ± 14.2	0.001
Sex, n (%)			
Male	8 (15.7)	30 (73.2)	<0.001
Female	43 (84.3)	11 (26.8)	

Primary outcome: ASIA score change

Mean ASIA score improved from 4.14 ± 0.84 preoperatively to 4.74 ± 0.66 postoperatively (p < 0.001, paired t-test) (Table 6). Of 93 patients, 49 (52.7%) improved, 43 (46.2%) remained stable (including 33 with baseline ASIA E), and 1 (1.1%) deteriorated (Table 7).

**Table 6:** Change in ASIA Impairment Scale Score from admission to last follow-up (N = 93). ASIA Impairment Scale grades were converted to numeric values (A = 1, B = 2, C = 3, D = 4, E = 5) for statistical analysis. Data represent all 93 patients with complete paired ASIA scores (admission and last follow-up). The p-value was calculated using a paired t-test to assess within-group change from admission to follow-up. ASIA: American Spinal Injury Association.

Time point	N	Mean	SD	25th	50th	75th
ASIA at admission	93	4.14	0.84	4	4	5
ASIA at last follow-up	93	4.74	0.66	5	5	5

**Table 7:** Individual patient changes in ASIA score (N = 93). Summary: Improvement 49 (52.7%), stable 43 (46.2%), deterioration 1 (1.1%). ASIA grades represent neurological function: A (complete – no motor/sensory function), B (incomplete - sensory only), C (incomplete - limited motor function), D (incomplete - functional motor ability), and E (normal function). The complete ASIA classification is provided in Table 1. ASIA: American Spinal Injury Association.

ASIA at Admission	A	B	C	D	E	Total
A	1	1	-	-	-	2
B	-	-	-	1	-	1
C	-	-	1	2	5	12
D	-	-	-	7	38	45
E	-	-	-	-	33	33
Total	1	1	2	13	76	93

Subgroup analyses

Effect of Medical Comorbidities

The presence of any medical comorbidity did not significantly influence ASIA score improvement when comparing patients with one or more comorbidities (n = 74) to those without any comorbidities (n = 19) (p = 0.37, between-group comparison) (Table 8). Both groups demonstrated significant within-group improvement from admission to last follow-up (p < 0.001 for each group).

**Table 8:** Change in ASIA score by presence of comorbidities (N = 93). p = 0.37 between groups; p < 0.001 for improvement within each group. ASIA: American Spinal Injury Association.

Comorbidities	N	ASIA admission (mean ± SD)	ASIA follow-up (mean ± SD)	p-value
None	19	4.32 ± 0.75	4.79 ± 0.54	0.37*
One or more	74	4.09 ± 0.86	4.73 ± 0.69	
Total	93	4.14 ± 0.84	4.74 ± 0.66	

**Figure 2:**
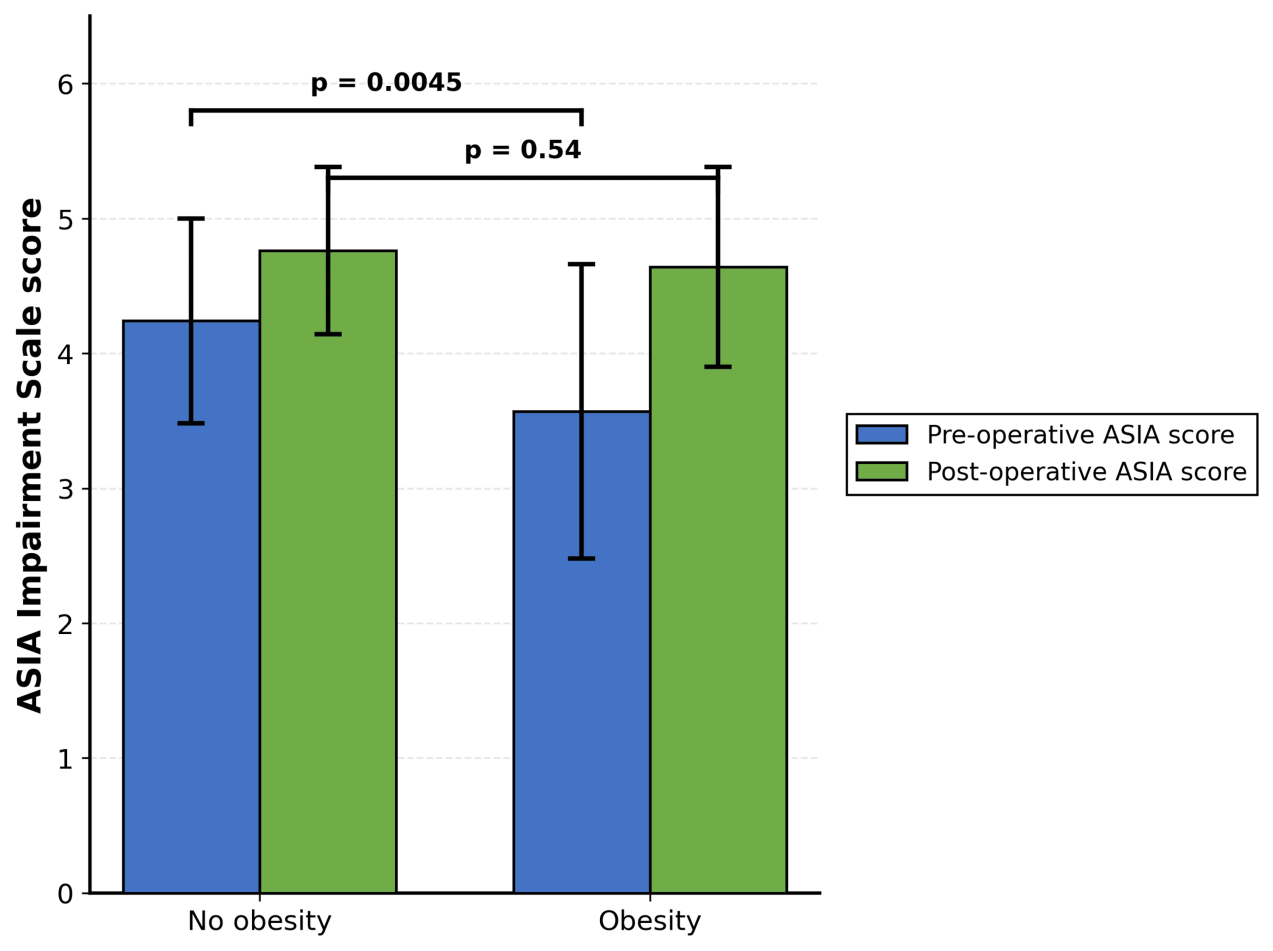
Comparison of ASIA scores between obesity and non-obesity groups. ASIA Impairment Scale scores at preoperative and postoperative time points stratified by obesity status. Patients without obesity (n = 79) demonstrated significantly higher preoperative ASIA scores compared to patients with obesity (n = 14) (4.24 ± 0.76 vs 3.57 ± 1.09, p = 0.0045). Following surgery, both groups achieved similar postoperative ASIA scores (4.76 ± 0.62 vs 4.64 ± 0.74, p = 0.54), indicating that the between-group difference in ASIA score change (p = 0.005) primarily reflected differences in baseline neurological status rather than differential surgical outcomes. Error bars represent SD. p < 0.01; NS: Not significant; ASIA: American Spinal Injury Association.

Analysis of specific comorbidities revealed that obesity was associated with a statistically significant between-group difference (p = 0.005), which was attributable to worse baseline neurological status in obese patients (ASIA admission score: 3.57 ± 1.02 in obese patients vs. 4.24 ± 0.77 in non-obese patients, p = 0.0045), rather than differential postoperative outcomes (ASIA follow-up scores were comparable: 4.64 ± 0.50 in obese vs. 4.76 ± 0.68 in non-obese, p = 0.54) (Table 9 and Figure 2). No other individual comorbidity demonstrated a significant effect on neurological outcomes.

**Table 9:** Change in ASIA score by specific comorbidities (N = 93). *p < 0.001 for improvement within all groups. ASIA: American Spinal Injury Association.

Comorbidity	ASIA admission (mean ± SD)	ASIA follow-up (mean ± SD)	p-value*
Cardiac disease			
Absent (n = 84)	4.14 ± 0.87	4.75 ± 0.67	0.087
Present (n = 9)	4.11 ± 0.60	4.67 ± 0.50	
Anticoagulation			
No (n = 77)	4.16 ± 0.81	4.75 ± 0.69	0.88
Yes (n = 16)	4.06 ± 1.00	4.69 ± 0.49	
Diabetes mellitus			
No (n = 70)	4.26 ± 0.77	4.80 ± 0.60	0.15
Yes (n = 23)	3.78 ± 0.95	4.57 ± 0.79	
Hypertension			
No (n = 41)	4.27 ± 0.81	4.73 ± 0.74	0.087
Yes (n = 52)	4.04 ± 0.86	4.75 ± 0.59	
Obesity			
No (n = 79)	4.24 ± 0.77	4.76 ± 0.68	0.005
Yes (n = 14)	3.57 ± 1.02	4.64 ± 0.50	
Smoking			
No (n = 78)	4.21 ± 0.71	4.78 ± 0.55	0.043
Yes (n = 15)	3.80 ± 1.32	4.53 ± 1.06	

Effect of Patient Sex

ASIA score change did not differ significantly between male patients (n = 38) and female patients (n = 55) (p = 0.52, between-group comparison). Both sexes demonstrated comparable improvement from admission to last follow-up (Table 10).

**Table 10:** Change in ASIA score by sex (N = 93). p = 0.52 between groups; p < 0.001 within each group. ASIA: American Spinal Injury Association.

Sex	N	ASIA admission (mean ± SD)	ASIA follow-up (mean ± SD)	p-value
Male	38	4.11 ± 0.80	4.76 ± 0.71	0.52
Female	55	4.16 ± 0.88	4.73 ± 0.62	
Total	93	4.14 ± 0.84	4.74 ± 0.66	

Effect of Tumor Pathology

ASIA score change did not differ significantly between patients with meningioma (n = 52, including 51 WHO grade I and 1 WHO grade II) and those with schwannoma (n = 41) (p = 0.61, between-group comparison). Both pathological types showed significant within-group improvement (p < 0.001 for each group) (Table 11).

**Table 11:** Change in ASIA score by pathology (N = 93). p = 0.61 between groups; p < 0.001 within each group.

Pathology	N	ASIA admission (mean ± SD)	ASIA follow-up (mean ± SD)	p-value
Meningioma	52	4.10 ± 0.85	4.73 ± 0.60	0.61
Neurinoma	41	4.20 ± 0.84	4.76 ± 0.73	
Total	93	4.14 ± 0.84	4.74 ± 0.66	

Effect of Symptom Duration

Symptom duration data were available for 89 of 93 patients. Among these 89 patients, complete paired ASIA scores (both admission and follow-up) were available for 88 patients; therefore, ASIA analysis by symptom duration was limited to these 88 patients. The one patient with symptom duration ≤1 month was excluded from comparative analysis due to insufficient sample size for that category. No significant difference in ASIA score improvement was observed between patients with symptom duration of 1 month to 1 year (n = 38) and those with symptoms lasting >1 year (n = 50) (p = 0.20, between-group comparison) (Table 12).

**Table 12:** Change in ASIA score by symptom duration (n = 88). p = 0.20 between groups. Symptom-duration information was documented for 88 of the 93 patients, which explains the sample size presented in this table.

Duration	N	ASIA admission (mean ± SD)	ASIA follow-up (mean ± SD)	p-value
1 month to 1 year	38	4.11 ± 0.80	4.82 ± 0.39	0.2
>1 year	50	4.16 ± 0.89	4.68 ± 0.82	
Total	88	4.14 ± 0.85	4.74 ± 0.67	

Effect of Tumor Location

Tumor location was analyzed by comparing each spinal region (cervical, thoracic, lumbar) against all other regions combined (control group). Cervical spine tumors (n = 25) showed no significant difference in ASIA score improvement compared with non-cervical tumors (n = 68) (p = 0.75, between-group comparison). Thoracic spine tumors (n = 47) showed no significant difference compared with non-thoracic tumors (n = 46) (p = 0.27, between-group comparison). Lumbar spine tumors (n = 26) showed a trend toward significance when compared with non-lumbar tumors (n = 67) (p = 0.059, between-group comparison), but this did not reach the conventional threshold for statistical significance (Table 13).

**Table 13:** Change in ASIA score by tumor location (N = 93). *p < 0.001 for improvement within all groups. Note: One patient had a sacral tumor (not shown, n = 1). ASIA: American Spinal Injury Association.

Location	N	ASIA admission (mean ± SD)	ASIA follow-up (mean ± SD)	p-value*
Cervical spine				
Yes	25	4.28 ± 0.46	4.92 ± 0.28	0.75
No	68	4.09 ± 0.94	4.68 ± 0.74	
Thoracic spine				
Yes	47	4.00 ± 0.96	4.68 ± 0.73	0.27
No	46	4.28 ± 0.69	4.80 ± 0.58	
Lumbar spine				
Yes	26	4.35 ± 0.85	4.73 ± 0.72	0.059
No	67	4.06 ± 0.83	4.75 ± 0.64	

Correlation With Continuous Variables

Spearman correlation analysis revealed no significant associations between the magnitude of ASIA score improvement and the following continuous variables: patient age at surgery (r = 0.158, p = 0.131), tumor size based on pathological measurements (r = -0.029, p = 0.783), operative duration (r = -0.176, p = 0.091), or total hospitalization duration (r = 0.142, p = 0.174) (Table 14).

**Table 14:** Correlation with continuous variables (N = 93). ASIA: American Spinal Injury Association.

Variable	Spearman’s r	p-value
Age at surgery (years)	0.158	0.131
Tumor size (cm³)	-0.029	0.783
Operative duration (min)	-0.176	0.091
Hospital stay (days)	0.142	0.174

Intraoperative Monitoring

Among the 60 patients for whom complete IONM data and paired ASIA scores (admission and follow-up) were available, 10 patients (16.7%) demonstrated signal impairment at the conclusion of surgery, while 50 patients (83.3%) showed no impairment at that time. When comparing ASIA score changes between these two groups, no statistically significant difference was observed (p = 0.76, between-group comparison) (Table 15). Both groups showed improvement in ASIA scores from admission to last follow-up.

**Table 15:** ASIA score by electrophysiological monitoring (n = 60). p = 0.76 between groups; p < 0.001 within each group. Analysis includes 60 patients with complete intraoperative neurophysiological monitoring (IONM) data and paired ASIA scores (admission and follow-up). Signal impairment at the conclusion of surgery was present in 10 patients (16.7%) and absent in 50 patients (83.3%). The p-value (p = 0.76) represents the between-group comparison. ASIA: American Spinal Injury Association.

Monitoring result	N	ASIA admission	ASIA follow-up	p-value
No impairment	50	4.25 ± 0.84	4.78 ± 0.50	0.76
Impairment present	10	3.90 ± 0.32	4.50 ± 0.97	
Total	60	4.20 ± 0.79	4.74 ± 0.60	

## Discussion

In this retrospective predictive study, we examined the feasibility of creating a model to predict the risk of neurological complications following surgery for meningioma or schwannoma resection from the spinal cord, based on preoperative patient characteristics and comorbidities. This study differs from previous investigations on this subject through its specific utilization of the ASIA classification system, broader pathology spectrum, and larger patient population. The ASIA scale, derived from the spinal trauma field, is well-established in that domain [21].

We examined multiple characteristics defined as potential risk factors for their capacity to predict neurological impairment following IDEM tumor resection surgery. Demographic characteristics, current disease parameters, and medical comorbidities were systematically evaluated.

Principal findings

For study purposes, patients’ ASIA scores were assessed before and after surgical intervention. A statistically significant improvement in ASIA score was demonstrated for all patients (p < 0.001), indicating overall neurological improvement following surgery.

Impact of medical comorbidities

Regarding medical comorbidities, of the 93 patients included in the study, 19 had no documented comorbidities while 74 had one or more comorbidities, with 4 patients having six concurrent comorbid conditions. No statistically significant difference in ASIA score change was identified between the 19 patients without comorbidities and the 74 patients with comorbidities (p = 0.37).

Upon examination of specific medical comorbidities, with the exception of obesity, no statistical significance was recorded in ASIA score change between patients with versus without each examined comorbidity. As demonstrated in this study, significance was recorded for obesity (p = 0.005). However, as illustrated in Figure 1 and discussed, this significance can be attributed to a lower preoperative ASIA score in the obesity group relative to the general population (p = 0.0045). Critically, after surgery there was no significant difference between groups (p = 0.54), suggesting that patients with obesity may actually experience proportionally greater improvement than the general population, ultimately achieving similar functional outcomes.

This result aligns with recent meta-analyses and large cohort studies in spine surgery, which show that while obesity is associated with increased perioperative risks such as higher rates of wound infection, venous thromboembolism, and longer operative times, neurological recovery and patient-reported functional outcomes are comparable between obese and nonobese patients when measured by validated scales, including ASIA and the Oswestry Disability Index [22]. Obesity does not independently predict poorer neurological improvement postoperatively, provided that surgical indications are met and perioperative management is optimized [12,23].

Impact of tumor pathology

Regarding tumor pathology, 52 patients had meningioma and 41 had schwannoma. Comparison of the two groups with respect to ASIA score change did not yield statistical significance (p = 0.61). Therefore, it can be concluded that for these two pathology types, neurological prognosis is favorable and comparable.

While pathology results are determined after surgery and thus cannot constitute a true preoperative risk factor assessment, tumor pathology can often be predicted preoperatively based on MRI imaging characteristics, which demonstrate distinct features for each tumor type, including the presence of a dural tail sign (highly specific for meningioma), cystic change (highly specific for schwannoma), and neural foraminal extension (more common in schwannoma). Meningiomas are more likely to be thoracic, smaller, and occur in older females, while schwannomas are more often lumbar, larger, and occur in younger patients. Multivariable analyses show that cystic change, dural tail sign, age, and lumbar location are independent predictors of pathology, with high interobserver agreement and diagnostic accuracy [24]. Scoring systems incorporating these features achieve strong diagnostic performance (AUC up to 0.87) [25].

Impact of demographic factors

Regarding demographic characteristics examined (age and sex), statistical significance was not demonstrated in ASIA score change analysis, with p-values of 0.174 and 0.52, respectively. This indicates that surgical outcomes are equivalent across age groups and between sexes.

Multiple studies support this finding. For age, large cohort analyses of spinal meningioma surgery demonstrate that older patients achieve neurological improvements comparable to younger patients, and age should not be considered a contraindication for surgery; functional and neurological recovery rates are similar regardless of age group [26,27]. For sex, while some differences in functional independence have been observed, these do not translate into significant differences in ASIA score improvement after adjusting for confounders; both males and females experience similar neurological recovery as measured by ASIA scores [28,29].

Impact of tumor size

In examination of tumor size as a risk factor for deterioration in ASIA score, no statistical significance was demonstrated (p = 0.91), suggesting that even larger tumors can be safely resected without increased risk of neurological decline.

This finding is supported by multiple studies showing that tumor length, volume, and occupation ratio are not independently associated with postoperative neurological deficits when controlling for other factors such as preoperative neurological status and tumor location [30].

While larger tumors may correlate with worse preoperative neurological function, the lack of significance in postoperative ASIA score change suggests that even larger tumors can generally be safely resected without increased risk of neurological decline [9]. The most robust predictors of postoperative neurological deterioration are preoperative neurological deficits and specific tumor locations (e.g., thoracolumbar junction), rather than tumor size itself [17].

Therefore, surgical decision-making should prioritize neurological status and anatomical considerations over tumor size alone when assessing risk for neurological complications after resection of intradural extramedullary spinal tumors.

Perioperative factors

Perioperative factors such as operative duration, hospitalization duration, and intraoperative electrophysiological monitoring results do not demonstrate a statistically significant association with changes in ASIA scores following surgical resection of intradural extramedullary spinal tumors (p-values 0.091, 0.174, and 0.76, respectively).

Current evidence indicates that while intraoperative neurophysiological monitoring (IONM), including motor evoked potentials (MEPs) and multimodal IONM, can accurately predict new motor deficits in some contexts, transient or non-significant changes in IONM are not reliably associated with long-term neurological deterioration in IDEM tumor surgery [31-33]. Operative duration and hospitalization duration are not independent predictors of neurological outcome; instead, preoperative neurological status and tumor characteristics are more relevant for prognosis [14,33].

Thus, these perioperative parameters are not significant risk factors for neurological deterioration as measured by ASIA score in the context of IDEM tumor resection, and their lack of statistical significance is consistent with the current clinical literature [14,31-33].

Study objectives achieved

The study’s first objective was to examine whether risk factors exist that can influence neurological complications following IDEM tumor (meningioma or schwannoma type) resection surgery. Based on the results of this study and the statistical analysis, no risk factors were identified that were associated with neurological impairment following surgery. Moreover, in all statistical tests performed, significant improvement was demonstrated in ASIA score change between preoperative and postoperative status across all examined subgroups of the population (p < 0.001 in all groups).

As mentioned, the study’s secondary objective was to demonstrate that these surgical procedures are safe from a neurological perspective. It is evident that across all examined groups, an increase in ASIA score was recorded with p < 0.001, leading to the conclusion that not only are neurological complications uncommon after these surgeries, but improvement in neurological functional capacity was also consistently demonstrated.

Comparison with previous literature

The neurological outcomes in this study, in which 52.7% of patients showed improvement, 46.2% remained stable, and only 1.1% experienced deterioration in ASIA scores after IDEM tumor resection, are highly favorable and compare well to, or slightly exceed, outcomes reported in previous literature that primarily used the McCormick scale and smaller cohorts. In large retrospective series using the McCormick scale, improvement rates after IDEM tumor surgery typically range from 46% to 54%, stability from 28% to 52%, and deterioration from 2% to 5% at early follow-up [9,34]. For example, a study of 121 spinal meningioma patients found 46% improved, 52% remained unchanged, and 2% worsened at discharge, with slightly higher improvement and deterioration rates at follow-up [9]. Another series of 60 thoracic IDEM meningiomas reported similar improvement and stability rates, with worse outcomes for ventral tumors [34].

The use of the ASIA scale in the current study provides a more granular and validated assessment of neurological function compared to the McCormick scale, which is less sensitive to subtle changes. The very low rate of deterioration (1.1%) in this cohort is at the lower end of the range reported in the literature, suggesting that surgical resection of IDEM tumors is overall safe and effective, with most patients experiencing improvement or stabilization of neurological status [9,34].

Limitations of the ASIA scale

The ASIA classification scale has several important limitations. It cannot reliably distinguish patients with isolated sensory impairment from those with no impairment, because the ASIA Impairment Scale (AIS) grade E is assigned only if both motor and sensory function are normal on examination, and individuals without spinal cord injury do not receive an AIS grade at all. Thus, patients with subtle or isolated sensory deficits may be misclassified or not captured by the scale [35].

Inter-rater variability in motor strength assessment can affect the assigned ASIA score, as the motor exam relies on manual muscle testing graded on a 6-point scale. Studies have shown that the lowest agreement among clinicians occurs in the determination of motor levels, with concordance rates as low as 62% for right and left motor levels, compared to higher agreement for sensory levels. This variability can lead to misclassification, particularly between AIS B and C grades, and underscores the need for rigorous examiner training and standardized technique [36].

Additionally, the ASIA scale may paradoxically show a worsening in AIS grade despite actual neurological improvement, especially in cases where motor or sensory changes do not align with the scale’s categorical thresholds [37]. These limitations highlight the need for careful interpretation of ASIA scores in both clinical and research settings.

Clinical implications

The clinical implications for patient counseling and informed consent, given that a systematic review of multiple criteria as potential risk factors for deterioration in ASIA score after intradural extramedullary (IDEM) tumor resection found no identifiable risk factors for neurological deterioration and demonstrated a high likelihood of neurological improvement or stability across diverse patient demographics and comorbidities, are that patients should be counseled that surgical resection of IDEM tumors is associated with a very high probability of neurological benefit and a very low risk of worsening, regardless of age, sex, comorbidities, or tumor size. This favorable risk profile is supported by large cohort studies and systematic reviews, which consistently show that most patients experience improvement or stability in neurological function postoperatively, with deterioration being rare [1,4,9,38].

No consistent preoperative, intraoperative, or perioperative risk factors for neurological decline have been identified, further supporting the safety and efficacy of surgery for IDEM tumors [31,39,40]. However, counseling should also include discussion of rare but possible complications, such as wound infection, CSF leak, or approach-related neurological deficits, particularly for anteriorly located thoracic tumors [6].

Patients should be informed that while IONM can help predict motor deficits, no single modality or clinical factor reliably predicts sensory decline or overall neurological deterioration, and that quality of life and patient-reported outcomes typically improve after surgery [31,32,38]. The limitations of current risk prediction tools, including the ASIA scale’s insensitivity to isolated sensory deficits and inter-rater variability, should be acknowledged during informed consent discussions.

Study limitations

This study has several limitations. The retrospective single-institution design limits generalizability and causal inference. Follow-up duration varied among patients (median 5 months, range 1-36 months), potentially affecting outcome assessment. While the ASIA scale provides standardized neurological assessment, it may not capture subtle changes in function. Intraoperative monitoring data were not documented for all patients (n = 60/93). Although our sample size (n = 93) exceeds most published series of IDEM tumors, statistical power remains limited for detecting small effect sizes in subgroup analyses, and the low rate of neurological deterioration (1.1%) precludes meaningful risk factor analysis for adverse outcomes.

Future directions

Prospective multicenter studies with larger sample sizes and standardized follow-up protocols are needed to validate these findings. Given the uniformly favorable outcomes observed, future research might focus on quality-of-life measures, functional independence scores, and long-term neurological outcomes to identify more subtle predictors of surgical benefit. Investigation of extended postoperative follow-up would provide valuable prognostic information for patient counseling.

## Conclusions

In this retrospective cohort, surgical resection of intradural extramedullary spinal tumors was associated with favorable neurological outcomes, with statistically significant improvement in ASIA scores following surgery. The majority of patients showed neurological improvement or remained stable, with deterioration being rare.

Among the preoperative and perioperative variables examined in this study, including patient demographics, medical comorbidities, tumor characteristics, and intraoperative parameters, none demonstrated statistically significant associations with postoperative neurological deterioration. Patients with medical comorbidities achieved outcomes comparable to those without comorbidities.

While the retrospective design and modest sample size limit our ability to identify rare risk factors or establish causal relationships, these findings suggest that surgical resection of IDEM tumors may be performed with favorable neurological outcomes across diverse patient populations. The systematic use of the ASIA Impairment Scale provides standardized assessment that may facilitate future comparative studies and meta-analyses.

These results support evidence-based patient counseling regarding the generally favorable neurological prognosis following IDEM tumor surgery, though prospective multicenter validation is warranted to confirm these findings and to identify potential predictors of the rare cases of neurological deterioration.
